# Carbonyl reductase 1 catalyzes 20β-reduction of glucocorticoids, modulating receptor activation and metabolic complications of obesity

**DOI:** 10.1038/s41598-017-10410-1

**Published:** 2017-09-06

**Authors:** Ruth A. Morgan, Katharina R. Beck, Mark Nixon, Natalie Z. M. Homer, Andrew A. Crawford, Diana Melchers, René Houtman, Onno C. Meijer, Andreas Stomby, Anna J. Anderson, Rita Upreti, Roland H. Stimson, Tommy Olsson, Tom Michoel, Ariella Cohain, Arno Ruusalepp, Eric E. Schadt, Johan L. M. Björkegren, Ruth Andrew, Christopher J. Kenyon, Patrick W. F. Hadoke, Alex Odermatt, John A. Keen, Brian R. Walker

**Affiliations:** 1 University/BHF Centre for Cardiovascular Science, The Queen’s Medical Research Institute, University of Edinburgh, Edinburgh, UK; 2 Royal (Dick) School of Veterinary Studies, University of Edinburgh, Edinburgh, UK; 3 Division of Molecular and Systems Toxicology, Department of Pharmaceutical Sciences, University of Basel, Basel, Switzerland; 4 Mass Spectrometry Core Laboratory, Wellcome Trust Clinical Research Facility, The Queen’s Medical Research Institute, University of Edinburgh, Edinburgh, UK; 5 School of Social and Community Medicine, University of Bristol, Bristol, UK; 6 PamGene International, Den Bosch, The Netherlands; 7 Department of Internal Medicine, Division Endocrinology, Leiden University Medical Center, Leiden, The Netherlands; 8 Department of Public Health and Clinical Medicine, Umeå University, 901 87 Umeå, Sweden; 9 The Roslin Institute, University of Edinburgh, Easter Bush Campus, Edinburgh, UK; 10 Department of Genetics and Genomic Sciences, Icahn Institute for Genomics and Multiscale Biology, Icahn School of Medicine at Mount Sinai, New York, USA; 11 Department of Physiology, Institute of Biomedicine and Translation Medicine, University of Tartu, Tartu, Estonia; 12 Clinical Gene Networks AB, Stockholm, Sweden; 13 Department of Cardiac Surgery, Tartu University Hospital, Tartu, Estonia; 14 Integrated Cardio Metabolic Centre, Department of Medicine, Karolinska Institute, Stockholm, Sweden

## Abstract

Carbonyl Reductase 1 (CBR1) is a ubiquitously expressed cytosolic enzyme important in exogenous drug metabolism but the physiological function of which is unknown. Here, we describe a role for CBR1 in metabolism of glucocorticoids. CBR1 catalyzes the NADPH- dependent production of 20β-dihydrocortisol (20β-DHF) from cortisol. CBR1 provides the major route of cortisol metabolism in horses and is up-regulated in adipose tissue in obesity in horses, humans and mice. We demonstrate that 20β-DHF is a weak endogenous agonist of the human glucocorticoid receptor (GR). Pharmacological inhibition of CBR1 in diet-induced obesity in mice results in more marked glucose intolerance with evidence for enhanced hepatic GR signaling. These findings suggest that CBR1 generating 20β-dihydrocortisol is a novel pathway modulating GR activation and providing enzymatic protection against excessive GR activation in obesity.

## Introduction

Carbonyl reductase 1 is a member of the short chain dehydrogenase/reductase family and is most commonly studied for its role in exogenous drug metabolism, particularly the conversion of chemotherapeutic drug doxorubicin to cardiotoxic danurubicin^1, 2^. Significant effort has gone into developing inhibitors of this enzyme which could be administered as an adjunct to doxorubicin therapy and thus reduce cardiac side effects^3–5^. There is also marked biological variation in expression of the CBR1 protein between ethnicities^6^ and following exposure to environmental agents such as cigarette smoke^7^ and flavonoids^8^. However the physiological role of this enzyme is unknown. Here we describe a novel role for CBR1 in glucocorticoid metabolism.

Glucocorticoids act through ubiquitous glucocorticoid receptors (GR) and cell-specific mineralocorticoid receptors (MR) to modulate, for example, fuel metabolism, inflammation and salt and water balance. Plasma glucocorticoid concentrations are controlled by the hypothalamic-pituitary-adrenal axis, which balances adrenal secretion of glucocorticoids against their clearance from the circulation by intracellular enzymes, predominantly active in the liver and kidney. These enzymes also modulate intracellular glucocorticoid concentrations independently of plasma concentrations, thereby conferring tissue-specific control of GR and MR activation. For example, in mineralocorticoid-responsive tissues such as the kidney and colon, MR are protected from exposure to the high-affinity ligand cortisol by 11β-hydroxysteroid dehydrogenase type 2 (11β-HSD2)^9^, which converts cortisol to inert cortisone; inhibition of 11β-HSD2 results in cortisol-dependent excessive MR activation and hypertension. In contrast, in glucocorticoid-responsive tissues such as liver and adipose, cortisol is regenerated from cortisone by 11β-HSD type 1 (11β-HSD1) thereby amplifying GR activation^10^; inhibition of 11β-HSD1 improves glucose tolerance in patients with type 2 diabetes^11^. Further modulation of receptor activation may be conferred by generation of glucocorticoid metabolites which retain activity at corticosteroid receptors. For example, hepatic 5α-reduction is the predominant clearance pathway for cortisol in humans but the product of this pathway, 5α-tetrahydrocortisol (5α-THF), is a selective GR modulator which may contribute to anti-inflammatory signaling^12^; inhibition of 5α-reductase type 1 results in glucose intolerance and liver fat accumulation, likely due to increased cortisol action in liver or skeletal muscle^13^. In humans and in rodent models, obesity is associated with tissue-specific dysregulation of cortisol metabolism, for example increased 5α-reductase activity and altered 11β-HSD1 activity^14^.

We embarked on an investigation of cortisol metabolism in domesticated horses, for whom obesity is a growing problem^15^ and discovered that the predominant metabolite of cortisol (F) in this species is 20β-dihydrocortisol (20β-DHF), which is increased in obesity. 20β-DHF has previously been identified in equine^16^ and human^17^ urine. Increased urinary excretion of 20β-DHF has been associated with Cushing’s disease^18^ and hypertension^19^ in humans. In this study we: dissected pathway producing 20β-DHF in horses, humans and mice; documented the enzyme responsible as carbonyl reductase 1 (CBR1); discovered that 20β-DHF modulates GR; and demonstrated the metabolic consequences of inhibiting CBR1.

## Results

### 20β-Dihydrocortisol is a metabolite of cortisol in horses and humans and its urinary excretion is increased in obesity

**Figure 1:**
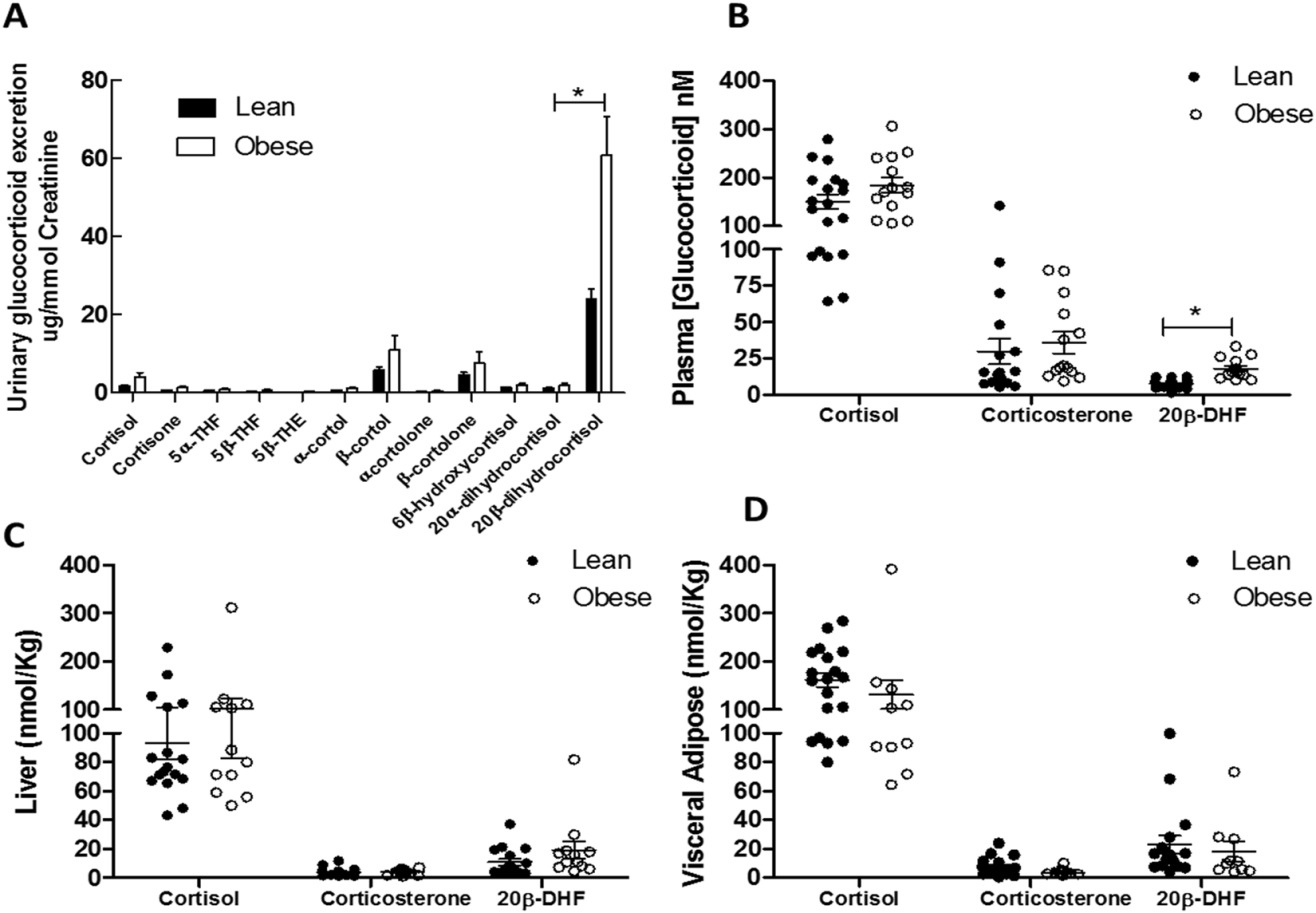
20β-Dihydrocortisol (20β-DHF) is an abundant cortisol metabolite which is increased in plasma and urine of obese horses. (**A**) Obese horses excreted significantly more urinary β-cortol, β-cortolone and 20β-DHF than lean horses as measured by GC-MS/MS. (**B**) Plasma 20β-DHF concentrations were significantly higher in obese horses compared to lean horses. (**C**) Hepatic 20β-DHF concentrations did not differ between lean and obese horses. (**D**) Visceral adipose 20β-DHF concentrations did not differ between lean and obese horses. Data are mean ± SEM, n = 14/group, *P < 0/05.

Urine, blood and tissue were collected from healthy (n = 14) and obese (n = 14) horses at post-mortem (see Supplementary Table S1 for clinical characteristics). Glucocorticoids were extracted and quantified using GC-MS/MS (urine) or LC-MS/MS (tissue and plasma). 20β-DHF accounted for approximately 60% of total glucocorticoid metabolite urinary excretion in healthy horses, and was increased in obese horses (Fig. 1A). Plasma 20β-DHF, but not cortisol, concentrations were also increased in obese horses (Fig. 1B). In visceral adipose tissue and liver, cortisol and 20β-DHF concentrations were measurable but not different between lean and obese horses (Fig. 1C–D).

**Figure 2:**
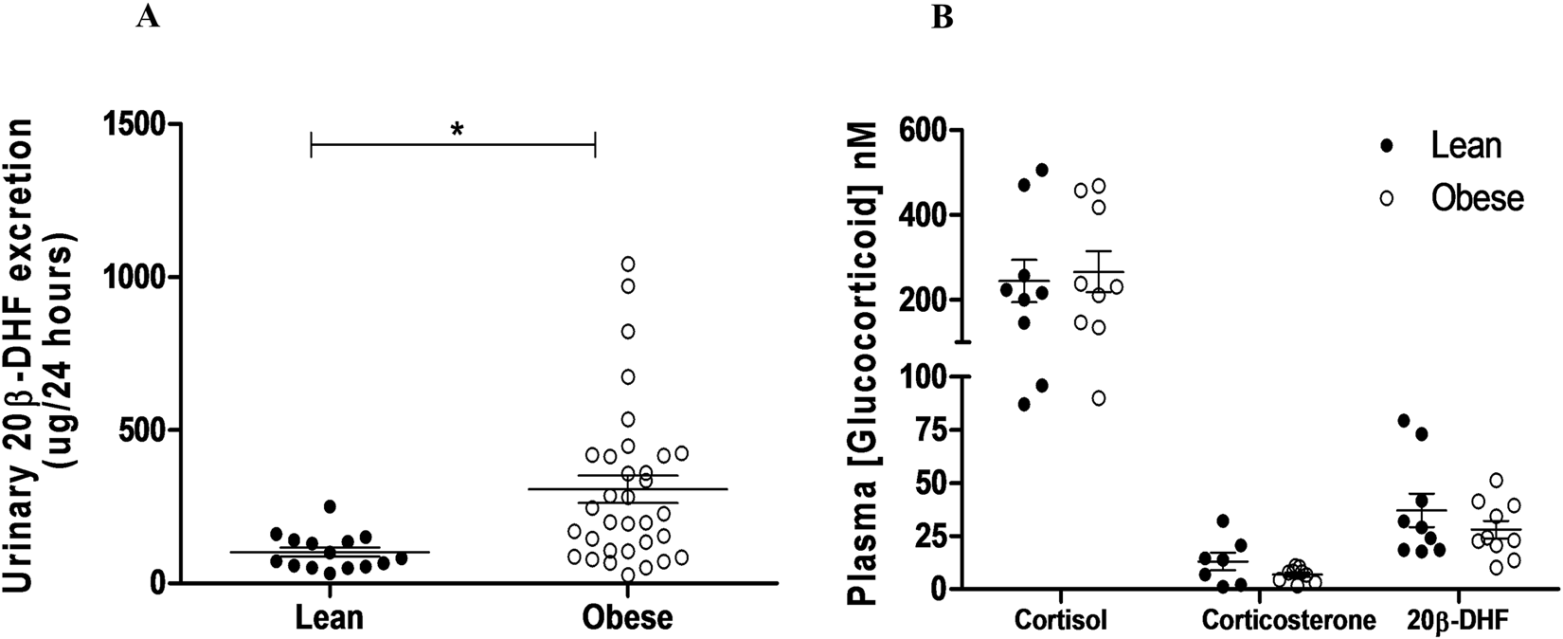
Urinary 20β-DHF is detectable in human urine and increased in obesity. (**A**) Obese (BMI >25, n = 37) humans excrete 20β-DHF at higher levels than lean (BMI <25, n = 15) humans. (**B**) 20β-DHF is readily detectable in human plasma but concentrations are not altered in obesity (n = 10/group). Plasma cortisol and corticosterone were not different between the groups. Data are mean ± SEM, *P < 0/05.

Twenty-four hour urine samples were collected from healthy lean men (mean age 37.7 ± 15.9 years), and from obese men with and without type 2 diabetes (mean age 51.1 ± 14.9 years). As previously reported, the human urinary cortisol metabolite profile was dominated by products of 5α- and 5β- reduction, β-cortol in particular^17^, and total metabolite excretion was increased in obesity^20^ (Supplementary Fig. S1). 20β-DHF was observed in human urine, accounting for approximately 3% of total urinary cortisol metabolites (Supplementary Fig. S1), and 20β-DHF excretion was increased in obesity (Fig. 2A), independently of the presence of diabetes, but was not disproportionately increased compared with other measured cortisol metabolites (see Supplementary Fig. S1 for metabolite pathways). 20β-DHF was also readily detected in plasma from healthy lean men at similar levels to corticosterone (Fig. 2B), but was not altered in obesity.

### Carbonyl reductase 1 converts cortisol to 20β-dihydrocortisol and is increased in equine, murine and human obesity

The enzyme responsible for 20β-DHF production was previously unknown. Carbonyl reductase 1 (CBR1) is a ubiquitously expressed short-chain dehydrogenase known for its role in xenobiotic metabolism^21^. Cortisol is reported as a substrate of CBR1 but its product has not been identified^21^. We found that recombinant human CBR1 converted cortisol to 20β-DHF in the presence of NADPH at a rate of 1.2 ± 0.4 ng/mg CBR1 protein per minute (1 μM cortisol substrate). Moreover, CBR1 accounts for equine production of 20β-DHF, which was the predominant metabolite in equine liver homogenate incubated with cortisol (Supplementary Fig. S2A), since this reaction was blocked by co-incubation with the CBR1 inhibitor quercetin in equine liver cytosol (Supplementary Fig. S2B). 20β-DHF was not produced by incubation of equine liver microsomes with cortisol.

**Figure 3:**
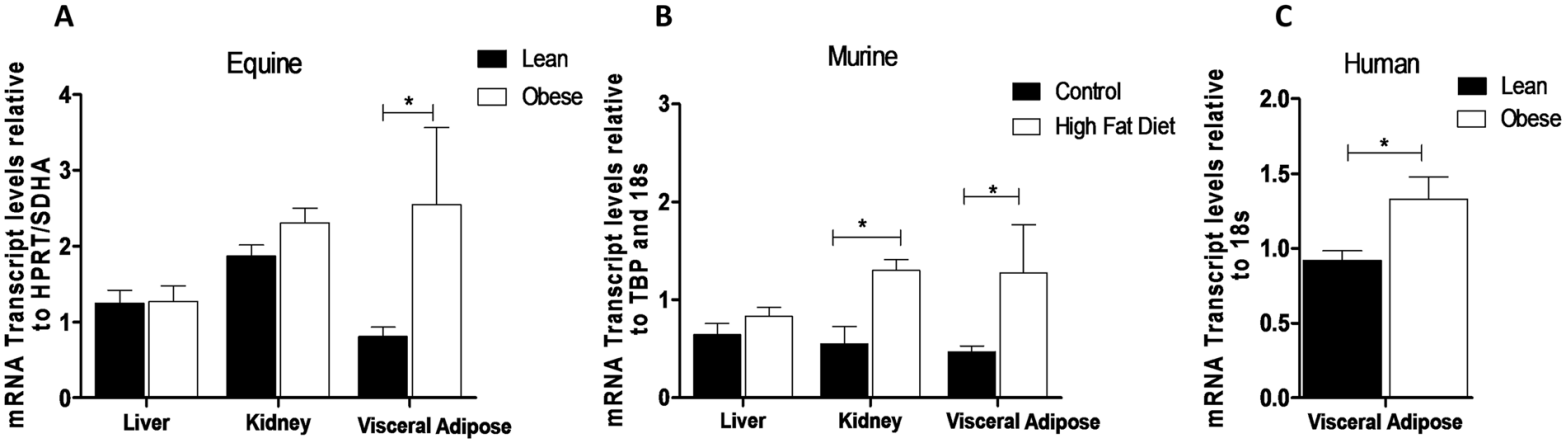
Carbonyl reductase 1 expression is increased in adipose tissue in obese horses, humans and mice. (**A**) *CBR1* mRNA transcript levels are increased in visceral adipose of obese horses (n = 14/group), (**B**) Visceral adipose *Cbr1* transcript levels were increased in mice on a high-fat diet for 6 weeks (n = 6/group). (**C**) Visceral adipose *CBR1* transcript levels were increased in obese humans (n = 8/group). Data are mean ± SEM, *P < 0.05.

*CBR1* is highly expressed in gut, liver, adipose and renal tissue of mice and humans (http://www.proteinatlas.org/ENSG00000159228-CBR1/tissue), the expression profile of horses has not been reported. We chose to examine the effect of obesity on expression of *CBR1* in liver and adipose tissue. Hepatic *CBR1* mRNA was not altered in obesity in horses or mice but *CBR1* mRNA was increased in adipose tissue of obese horses (Fig. 3A). *CBR1* mRNA was also higher in high-fat fed mice (Fig. 3B) and in visceral adipose tissue from obese compared with lean men (n = 8/group, Fig. 3C).

### Common functional genetic variants in the CBR1 locus predict metabolic disturbances in obesity

We used an expression quantitative trait loci (eQTL) approach in the STARNET dataset^22^ to test whether any SNPs in the *CBR1* locus had a functional effect on hepatic or visceral adipose CBR1 expression, and then tested their association with phenotypic traits in publicly accessible datasets using MR-Base^23^. There were no eQTLs which influenced visceral adipose expression of CBR1 but eQTLs were identified in liver. Further analyses suggested that SNPs associated with higher CBR1 expression in the liver were causally associated with higher fasting glucose (beta 0.01, se <0.01, p = 0.02), higher glycated haemoglobin (beta 0.01, se <0.01, p = 0.01) (Supplementary Table S5). There was no evidence that CBR1 expression was causally associated with fasting insulin, HOMA-B or HOMA-IR (p > 0.2), although there was suggestive evidence that higher CBR1 expression causes lower body fat (beta −0.01, se 0.01, p-0.06). In addition to these observations in population based cohorts, eQTLs of CBR1 in liver were associated with BMI in the STARNET study participants (rs2835288, p = 5.7E-4). The eQTL rs2835288 had a negative effect on BMI (r = −0.13) but a positive effect on CBR1 liver expression (r = 0.43); accordingly CBR1 liver expression and BMI were negatively correlated (r = −0.093, p = 0.03). Using a conservative causal inference test^24^ there was suggestive evidence (p = 0.07) that expression of CBR1 in liver was causal for variation in BMI.

### 20β-DHF activates glucocorticoid receptors

Given apparently contradictory associations of genetically high CBR1 activity with metabolic dysfunction but not obesity in humans, and the association of CBR1 expression and activity with obesity in multiple species, we investigated the interaction of 20β-DHF with GR in order to predict consequences of elevated CBR1 for GR activation.

**Figure 4:**
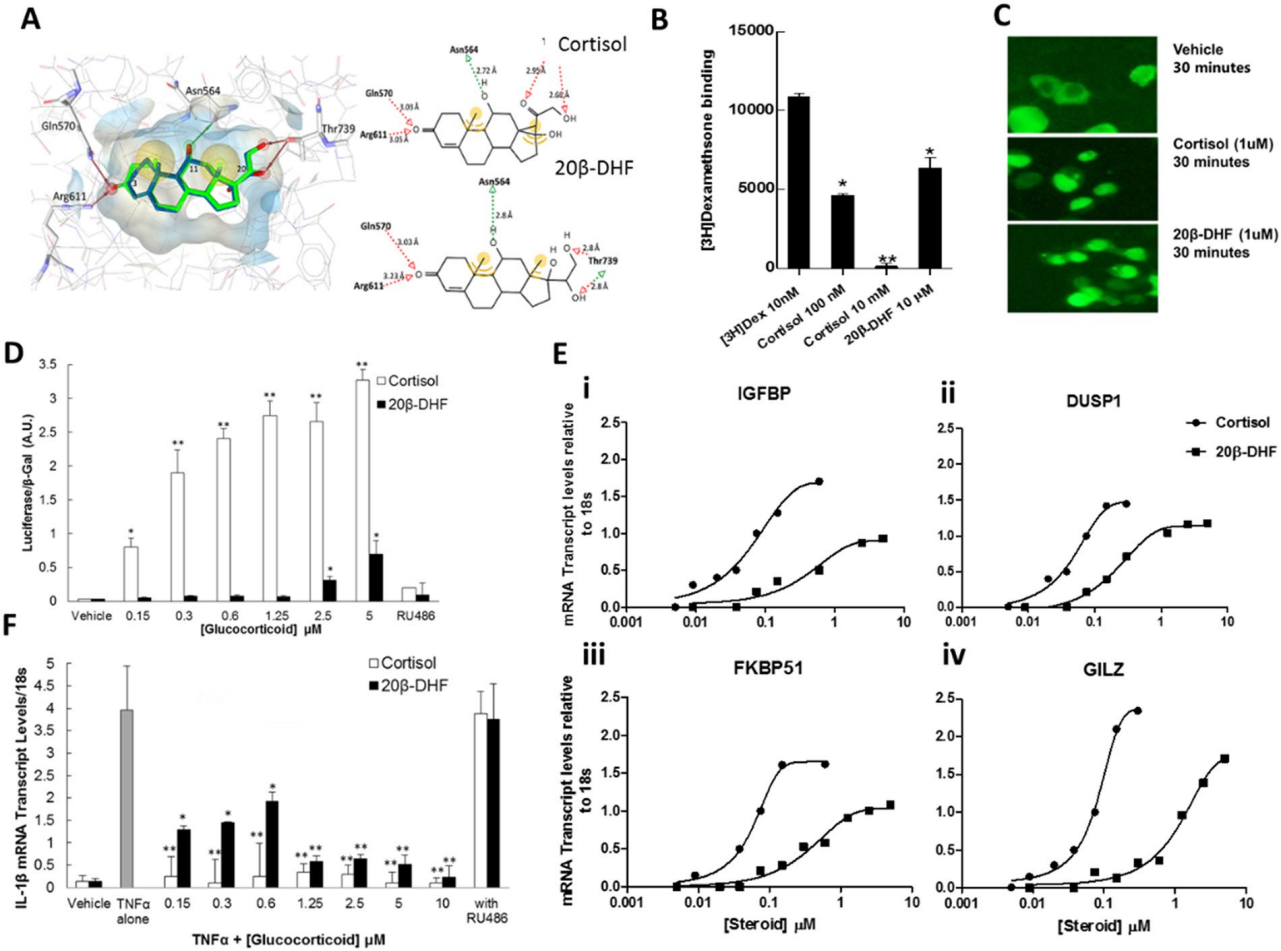
20β-Dihydrocortisol binds, translocates and activates glucocorticoid receptor inducing gene transcription and suppressing inflammatory gene transcription. (**A**) Docking of cortisol and 20β-DHF into the ligand binding site of GR. The automatically created pharmacophore indicates the essential structural features for ligand binding (red and green arrows with spheres display hydrogen-bond (H-bond) interactions and yellow spheres hydrophobic interactions). Amino acid residues crucial for ligand binding are shown as sticks. Compared to the binding interactions of cortisol 20β-DHF differs only in the hydroxyl group at the position 20, representing a H-bond donor instead of the carbonyl group of cortisol serving as H-bond acceptor. (**B**) Unlabelled 20β-DHF displaced ^3^[H]-dexamethasone from GR in the lysate of SF9 cells expressing GR. (**C**) 1uM 20β-DHF induced translocation of cytoplasmic GR to the nucleus of HEK293 cells within 30 minutes visualised by fluroescence imaging at 20x magnification. (**D**) 2.5uM 20β-DHF induced luciferase activation in A549 cells transfected with glucocorticoid responsive plasmid MMTV-luc. (**E**) 20β-DHF induced transcription of GR-responsive genes IGFBP1 (EC_50_ 0.51 µM), DUSP1 (EC_50_ 0.32 µM), FKBP51 (EC_50_ 0.44 µM) and GILZ (EC_50_ 1.25 µM) in A549 cells. (**F**) TNFα induced transcription of IL-1β in A549 cells, this was inhibited by cortisol and by 20β-DHF at 0.15 µM. Transcription was not reduced by co-incubation of cortisol or 20β-DHF with the GR antagonist RU486. Experiments were performed in triplicate on three occasions. Data are mean ± SEM (N = 3). Data were compared by two-way ANOVA and Bonferroni correction test: *P < 0.05, **P < 0.01 compared to vehicle.

Computational evaluation of the interactions formed by 20β-DHF with the GR ligand binding site using docking calculations revealed a similar binding pose compared to cortisol (Fig. 4A). Both ligands formed hydrogen-bonds (H-bonds) similar in length with the same amino acid residues (Arg611, Gln570, Asn564 and Thr739). The only difference observed was the hydroxyl group of 20β-DHF at position 20 representing a hydrogen bond donor instead of the carbonyl group of cortisol at the same position serving as hydrogen bond acceptor. Human epithelial A549 cells expressing endogenous GR and SF9 and HEK293 cells transfected with human GR were used to investigate 20β-DHF as an endogenous ligand of GR. In binding studies, unlabeled 20β-DHF displaced dexamethasone from GR in SF9 cell lysate preparations but only at 1000-fold higher concentration than cortisol (Fig. 4B). Nonetheless, transfection of HEK293 cells with GFP-GR showed that 20β-DHF induced nuclear translocation of GR within 30 minutes (Fig. 4C).

In functional studies, 20β-DHF was a weak agonist of GR. In A549 cells MMTV promoter-induced luciferase activity, indicative of GR activation, was only partially induced by 20β-DHF at high concentration (2.5 μM; Fig. 4D). However, endogenous GR-responsive genes glucocorticoid-induced leucine zipper (*GILZ*), insulin-like growth factor binding protein 1 (*IGFBP1*), dual specificity phosphatase 1 (*DUSP1*) and FK506-binding protein 51 (*FKBP51*) were all up-regulated by 20β-DHF in a concentration-dependent manner (Fig. 4E) and to a similar maximum as cortisol, albeit at substantially higher concentrations than cortisol. Similar dose-response relationships were seen comparing the effects of cortisol and 20β-DHF in preventing IL-1β induction by TNFα in A549 cells (Fig. 4F).

**Figure 5:**
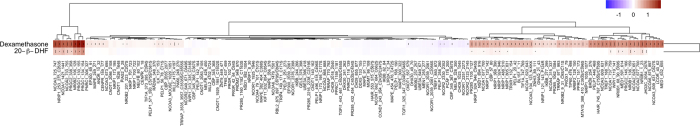
20β-Dihydrocortisol induces similar co-regulator interactions with GR as dexamethasone MARCoNI analysis of co-activator recruitment showed that on binding 20β-DHF, GR recruited 36% of the co-regulators recruited by dexamethasone. The colour of the bar represents the modulation index i.e. compound induced log-fold change of binding, red a positive fold change and blue a negative fold change. *P < 0.05, **P < 0.01, ***P < 0.001 compared to the unbound receptor.

Co-regulator recruitment by GR on binding 20β-DHF was assessed by microarray assay for real-time co-regulator-nuclear receptor interaction (MARCoNI) with the GR agonist dexamethasone used as a positive control^25^. Under these conditions, 20β-DHF-activated GR recruited approximately 36% of the co-regulators recruited by dexamethasone (Fig. 5 and supplementary Excel file).

### Pharmacological inhibition of Cbr1 in mice results in increased hepatic GR activation and worsens the metabolic effects of high-fat feeding

Knowing that both cortisol and 20β-DHF might amplify GR activation, we sought to test the effects of Cbr1 inhibition in mice to determine whether increasing the substrate/product balance would increase or decrease GR activation. Unlike horses and humans, mice produce corticosterone (B) rather than cortisol as their major glucocorticoid. To validate the use of murine models to study the CBR1/20β-dihydroglucocorticoid pathway, preparatory work included demonstration that 20β-dihydrocorticosterone (20β-DHB), the murine equivalent of 20β-DHF, induced MMTV-luciferase activity in HEK293 cells transfected with murine GR (Supplementary Fig. S3) and is present in murine plasma and tissue (Supplementary Fig. S4), and that *Cbr1* mRNA was higher in adipose of C57BL/6 J adult male mice fed on a high fat diet for 6 weeks than controls on a normal chow diet (n = 6/group) (Fig. 3B). Murine diet-induced obesity was therefore used as a model in which to investigate the functional role of CBR1 and 20β-dihydro metabolites. Groups of adult male C57BL/6 J mice (n = 12/group) maintained on a high fat diet were randomly assigned to groups receiving vehicle (ethanol) or Cbr1 inhibitor (quercetin, 50 μg/mouse/day, administered in drinking water for 6 weeks).

**Figure 6:**
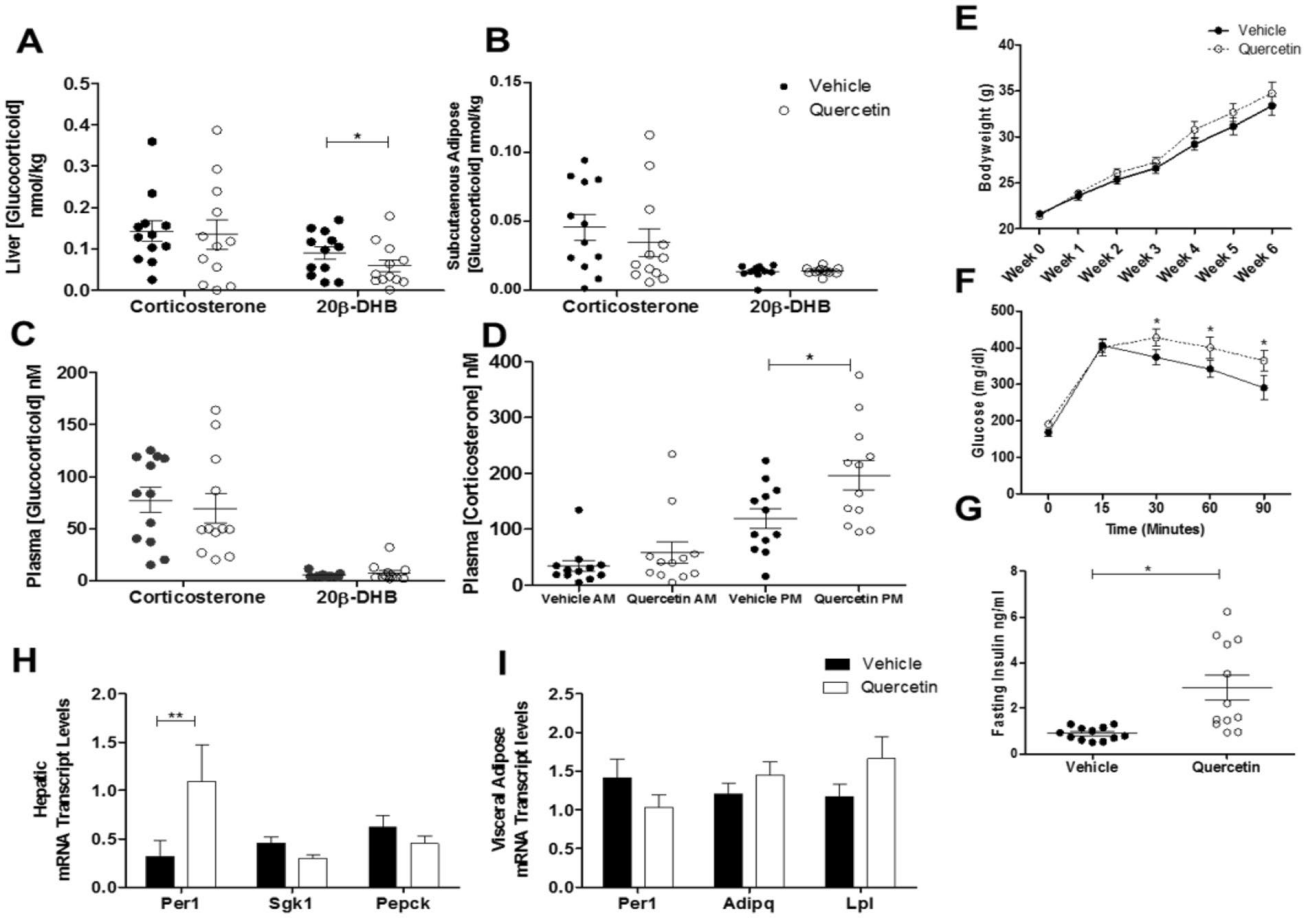
Inhibition of CBR1 in a murine model of diet-induced obesity results in increased GR activation and metabolic dysfunction. (**A**) Hepatic 20β-DHB levels were significantly lower in quercetin treated mice, (**B**) 20β-DHFB levels were not altered in subcutaneous adipose tissue, (**C**) plasma 20β-DHB levels were not altered by quercetin treatment (**D**) quercetin treatment resulted in increased peak plasma corticosterone levels. (**E**) Bodyweight was not different between mice in the vehicle treated group and mice treated with quercetin. (**F,G**) Quercetin-treated mice were significantly more insulin resistant and had higher fasted plasma insulin concentrations. (**H**) mRNA transcript levels of hepatic Per1 were increased in quercetin treated mice, (**I**) Adipose mRNA expression of Per1, Adiponectin (*Adipq*) and lipoprotein lipase (*Lpl*) were not altered by quercetin treatment. Data are mean ± SEM, n = 12/group, *P < 0.05.

Quercetin lowered hepatic 20β-DHB (Fig. 6A) and increased the ratio of Cbr1 substrate (corticosterone) to product (20β-DHB) in liver (vehicle B: 20β-DHB ratio 0.5 ± 0.2 versus quercetin B: 20β-DHB ratio 1.6 ± 0.4, P = 0.01). Quercetin did not alter 20β-DHB levels in subcutaneous adipose tissue (Fig. 6B) or plasma (Fig. 6C). Quercetin also raised peak plasma corticosterone concentrations (Fig. 6D) but did not affect food or water intake or bodyweight over the course of the experiment (Fig. 6E). However, quercetin raised fasting plasma insulin concentrations and blood glucose during glucose tolerance tests (Fig. 6F,G). Quercetin also increased hepatic expression of the GR-responsive gene Period 1 (*Per1)*, but did not alter the mineralocorticoid-responsive gene serum glucocorticoid kinase 1 (*Sgk1*) or key gluconeogenic enzyme phosphoenolpyruvate carboxykinase *(Pepck)* in the liver (Fig. 6H). Transcript levels of *Per1*, adiponectin and lipoprotein lipase were not altered by quercetin in subcutaneous adipose tissue (Fig. 6I).

## Discussion

We describe a novel pathway of glucocorticoid metabolism, whereby cortisol is converted to 20β-dihydrocortisol by the cytosolic enzyme CBR1, producing a metabolite which is a weak activator of GR. This pathway is up-regulated in adipose-tissue of obese horses, humans and mice; genetic variation in the liver predicts glucose dysregulation and its pharmacological inhibition alters the hypothalamic-pituitary-adrenal axis and tissue steroid levels in mice, with associated changes in GR-dependent gene expression and in metabolic homeostasis. This provides important new insights into the control of tissue glucocorticoid action and its contribution to cardiometabolic disease.

The glucocorticoid metabolite profile of horses, a cortisol-dominant species^26^, has not previously been described, although inter-species variation in hepatic cortisol metabolism has been reported^27^. 20β-DHF has been measured in horse urine^16^ and purported to be a sensitive indicator of cortisol administration, but ours are the first data showing 20β-DHF relative to other metabolites, and the first demonstrating 20β-DHF in plasma, adipose tissue and liver of horses. Predominance of 20β-DHF production occurs in other large herbivores, including sheep^28, 29^. The human cortisol metabolome is more thoroughly described^17, 30^, and is dominated by products of 5α- and 5β-reduction. However, 20β-DHF has previously been identified in human urine^17, 18^ at levels similar to that of 5α-tetrahydrocortisol (5α-THF). Occasional case reports indicate urinary excretion of 20β-DHF is increased in human Cushing’s syndrome^18^, collagen disease^31^, rheumatoid arthritis^32^, hypertension^19^ and liver cirrhosis^33, 34^. In our study 20β-DHF was readily detectable in the plasma of healthy humans at levels equal or higher to that of corticosterone, but 20β-DHF represented a much smaller proportion of cortisol metabolism than in horses.

There are reports of reduction of cortisol to 20β-DHF in various human cell/tissue types including kidney and prostate^35^, gingiva^36^, fibroblasts^37^ and thrombocytes^38^. We found that CBR1, a ubiquitously expressed member of the short-chain dehydrogenase/reductase (SDR) superfamily^1^ catalyzes the conversion of cortisol to 20β-DHF. *CBR1* expression is highest in tissues involved in detoxification or clearance, e.g. liver, colon, renal tubules and placenta^39^ and has been studied for its role in drug metabolism^3^ and as an antioxidant^40^. Its expression is associated with cancer, particularly lung cancer^7^, and reported to protect against pancreatic islet cell death^41^. Inhibitors of CBR1 have been proposed for use with chemotherapeutic agents to reduce the cardiotoxic side-effects of drugs such as doxorubicin^4^. Although glucocorticoids are known to be substrates of CBR1, the products have not been identified previously^40^. We attribute 20β-dihydro glucocorticoid generation to CBR1 since isolated CBR1 converts cortisol to 20β-DHF and not to other metabolites, hepatic microsomal preparations are devoid of such activity, and inhibition of CBR1 is sufficient to prevent 20β-DHF generation in equine liver cytosol and to lower tissue 20β-DHB in mouse liver *in vivo*.

The majority of cortisol metabolites are thought to be inert and are produced to facilitate steroid excretion. Some metabolites, however, such as 5α-tetrahydrocorticosterone (5α-THB) bind and activate GR^12^. Given that 20β-DHF was found in plasma and tissues of humans at similar levels to the endogenous glucocorticoid corticosterone, and is thus potentially of physiological significance, we investigated the action 20β-DHF on GR. We found that 20β-DHF bound GR with a lower affinity than that of cortisol but induced nuclear translocation of the receptor within 30 minutes, a time period comparable with cortisol. 20β-DHF induced transrepressive and transactivation effects after binding to GR albeit at higher concentrations than cortisol. The consequences of variation in CBR1 activity for GR activation are therefore hard to predict.

In human obesity, increased total cortisol production^42^ without consistently elevated plasma cortisol concentrations has been attributed to enhanced clearance of cortisol^20^, and in turn to increased 5α- and 5β-reductase and reduced 11β-HSD1 activities^20^. In humans, horses and mice obesity was associated with increased *CBR1/Cbr1* expression in adipose tissue, in horses with increased 20β-DHF in plasma, and in humans and horses with increased 20β-DHF in urine. Although 20β-DHF was not disproportionately raised in urine, total cortisol metabolite excretion was increased so these data are consistent with the CBR1/20β-DHF pathway contributing to increased cortisol clearance in obesity. Up-regulation of the CBR1 pathway in obesity was evident in adipose but not liver in horses and mice. In addition we did not identify any eQTL for *CBR1* expression in adipose suggesting that this up-regulation is a functional response to obesity. This is consistent with intra-adipose inflammation and hypoxia in obesity, since *CBR1* expression is up-regulated in response to hypoxia and inflammation via transcription factors including Nrf2, AhR and HIF-1α^43, 44^. In contrast our data suggest that there are genetic influences on hepatic *CBR1* expression in humans and that higher expression is associated with higher leptin, higher fasting glucose and higher HbA1c. Tissue-specific regulation of *CBR1* is reported^43, 45, 46^ so the eQTLs we identified may, for example, exert their influence through liver-specific promoter(s).

To further explore the contribution of CBR1 dysregulation in obesity, we administered the Cbr1 inhibitor quercetin to mice with diet-induced obesity. Pharmacodynamic data suggest that quercetin inhibited corticosterone conversion to 20β-DHB in liver, but not in adipose tissue; this may indicate that adipose 20β-DHB is mainly derived from plasma rather than from local generation, or that the drug was unable to penetrate adequately into adipose tissue. Although an eQTL predicting higher hepatic CBR1 was associated with adverse metabolic indices, a paradoxical deterioration in glucose metabolism was observed when we inhibited the enzyme. This was accompanied by altered GR-regulated genes in liver which may be explained by the effect of substrate (corticosterone) accumulation or by secondary activation of the HPA axis with elevated peak plasma corticosterone resulting from impaired clearance. A similar phenotype and liver transcript profile occurs in mice with genetic deletion of the glucocorticoid-inactivating enzyme 5α-reductase type 1^47^. Alternatively we could infer that there is a non-linear relationship between *Cbr1* expression and effect and that an optimal cortisol/20β-DHF balance may be required for normal liver GR activation, such that dysregulation of *Cbr1* in either direction leads to GR excess.

These findings are important since there are wide variations in CBR1 activity between individuals, in disease and after consumption of a number of naturally occurring CBR1 inhibitors: human tissue CBR1 expression and activity varies significantly between ethnic groups^6^; CBR1 expression and activity is increased in Down’s syndrome due to the location of the CBR1 gene on chromosome 21^48^; CBR1 inhibitors such as flavonoids and polyphenols are present in many foods and supplements^49^ and reported enhancers of CBR1 activity include components of cigarette smoke^7^. Our data suggest the resulting variation of CBR1/20β-DHF has important consequences for glucocorticoid metabolism and GR activation in health and disease.

## Materials and Methods

### Study design

We conducted case-control, cross-sectional or intervention studies in horses, humans, cells and mice. Sample sizes were chosen for 80% power to detect magnitudes of difference inferred from pilot data with the number of subjects and outcomes defined below or in figure legends. *In vitro* experiments were performed in triplicate with the number of experiments and outcomes defined below and in figure legends. Details on inclusion and exclusion criteria for horse and human subjects are detailed below. There were no dropouts and no outliers were excluded.

### Cortisol metabolism in horses

The first aim of the study was to characterize cortisol metabolism in lean and obese horses. We addressed this aim using an observational case-control study recruiting lean horses and obese horses that were destined for euthanasia at the Royal (Dick) School of Veterinary Studies, University of Edinburgh. Studies in horses were approved by the Royal (Dick) School of Veterinary Studies Ethics and Research committee (VERC 7014). The study was performed according to the approved ethical guidelines. The sample size (n = 14/group) was determined by interim analysis using total glucocorticoid metabolite excretion as the end-point (80% power to detect a 20% difference in groups, p < 0.05). Lean (body condition score, measure of obesity ≤3/5^50^) and obese (body condition score ≥4/5) castrated male and female horses destined for euthanasia, were recruited from clinics at the Royal (Dick) School of Veterinary Studies. Horses were excluded if they were less than 1 year old, suffering from any concurrent systemic illness or had received glucocorticoid treatment in the 3 months prior to commencement of the study. Blood was obtained after overnight fasting, between 0900 h and 1100 h, via an intravenous cannula inserted in the jugular vein for the purpose of euthanasia.

Horses were humanely euthanased (0900 h to 1100 h) with quinalbarbitone sodium and cinchocaine hydrochloride (1 mL/10Kg bodyweight; Somulose, Dechra Veterinary Products, Shrewsbury, UK). Samples of peri-renal adipose, liver and urine were snap frozen and stored at −80 °C.

Glucocorticoids were extracted from plasma, adipose and liver and quantified by LC-MS/MS (see supplementary methods). Urinary glucocorticoids were derivatized and quantified by GC-MS/MS (supplementary methods). Urinary creatinine was measured by the modified Jaffe’s reaction (IL650 analyzer, Instrumentation Laboratories). Glucocorticoid concentrations were expressed as μg/mmol creatinine. RNA was extracted from adipose and liver samples for quantification of *CBR1* mRNA relative to housekeeping genes SDHA and 18 s (see supplementary methods and supplementary Table S7).

### 20β-DHF in humans

In order to determine the relevance of 20β-DHF to human health and disease samples were collected from male participants at the University of Edinburgh with approval from the University of Edinburgh Research Ethics Committee, National Health Service Lothian Research and Development Office, and at Umea University with approval from the Umea Regional Ethical Review Board. The study was performed according to the approved ethical guidelines. Participants were required to give written informed consent prior to recruitment to the study. Lean individuals were defined as having a BMI <25 kg/m^2^ and obese individuals as having a BMI >25 kg/m^2^. Clinical details are given in supplementary Tables 2–4.

Twenty-four hour urine samples were obtained from healthy lean (n = 15) and obese (n = 18) men recruited as part of a separate study^13^. In addition, urine was collected from obese men (n = 19) with Type 2 diabetes (with no insulin treatment) (Supplementary Table S2). Morning fasted plasma samples were collected from healthy lean (n = 10) and obese (n = 10) men (Supplementary Table S3). Adipose biopsy samples for RNA extraction were collected from lean (n = 8) and obese (n = 8) individuals undergoing surgery (Supplementary Table S4). Glucocorticoids were extracted and quantified from plasma and urine as detailed in supplementary methods.

### CBR1 activity *in vitro*

In order to determine if CBR1 could convert cortisol to 20β-DHF recombinant human CBR1 (Source Bioscience, Nottingham, UK) was incubated with cortisol (1 mM) and NADPH (2 mM) for a time course (5, 10, 20, 30, 60 and 120 minutes) at 37 °C. The reaction was stopped with the addition of acetonitrile (500 µL). Deuterated cortisol (9, 11, 12, 12-[^2^H]_4_-cortisol) was added as an internal standard for quantification of cortisol and 20β-DHF. Following centrifugation (5 minutes) the supernatant was removed, dried down and re-suspended in mobile phase (60 µL 50:50 Methanol: water) for analysis by LC-MS/MS (Supplementary methods).

### Interrogation of genetic data for CBR1 expression and phenotypic associations

No genetic variants have been robustly associated with CBR1 enzyme activity. Therefore, genetic variants that are associated with CBR1 transcript levels in liver were used as a surrogate for CBR1 enzymatic activity. Expression quantitative trait loci (eQTLs) located near to the CBR1 gene and associated with CBR1 expression in the liver were identified from the Stockholm-Tartu Atherosclerosis Network Engineering Task (STARNET) study. STARNET comprises data on 600 cases of cardiovascular disease undergoing surgical intervention with collection of multiple tissue types including the liver. Genome wide genotyping and tissue expression analyses including RNAseq have been performed^22^.

A two-sample Mendelian randomization approach was used to estimate the effect of CBR1 expression in the liver on outcomes of body mass index (BMI), body fat, and glucose and insulin sensitivity. The outcome data were extracted from publicly available datasets, including from GIANT and MAGIC consortia, using MR-Base^23^. In cases where outcome data were available from more than one study, the study containing all the relevant information with the largest sample size was selected. The causal effect of CBR1 expression on the relevant outcomes was estimated using the Wald (or ratio) method. This method divides the coefficient from regression of the outcome on the genetic variant by the coefficient from regression of the exposure on the variant^51^; the former was derived from publicly available data and the latter from STARNET. This approach makes the assumption that all instrumental variables are valid and not subject to horizontal pleiotropy where a genetic variant affects the outcome via more than one biological pathway. Study overlap is a concern when undertaking two sample Mendelian randomization analyses. The STARNET study has not provided data to the GIANT or MAGIC consortia.

### 20β-DHF interaction with glucocorticoid receptor

#### Docking of 20β-DHF with GR

Docking studies were performed using the GOLD software version 5.2 (Cambridge Crystallographic Data Centre, Cambridge, UK)^52^. This software allows the identification of precise docking poses for small molecules in the binding pocket of a protein applying a genetic algorithm. The crystal structures with the Protein Data Bank (PDB) entry 4P6X [DOI:10.2210/pdb4p6x/pdb] was selected for GR. First the respective co-crystallized ligand, cortisol for GR, was removed from the binding pocket and re-docked into the binding site to examine whether GOLD could restore the original binding pose and therefore to validate the docking settings (RMSD value of 0.409 for GR). The GR binding sites were defined by the ligand surrounded by a 6 Å region lining the active site. GoldScore was used as scoring function.

Protein ligand interactions determined by the docking software were further assessed using LigandScout 3.12 (inte:ligand GmbH, Vienna, Austria). Based on chemical functionalities, geometric distances and angles between adjacent structures, this software automatically evaluates the observed binding pattern between the protein and the docked ligand^53^.

#### Glucocorticoid binding in SF9 cell lysates

Competitive GR binding experiments were conducted as described previously^54^. Briefly, recombinant human GRα baculovirus stock was produced using the Bac-to-Bac expression system and subsequently expressed in Sf9 cells according to the instructions by the manufacturer (Invitrogen, Carlsbad, CA). Sf9 cell lysates expressing recombinant human GR were then incubated in the presence of 10 nM [1,2,4,6,7–3 H]-dexamethasone and unlabelled competitor (either 10 μM or 100 nM cortisol or 10 μM 20β-DHF) for 4 h at 16 °C. Unbound ligand was separated by adding 5% dextran coated charcoal, followed by incubation at 4 °C for 10 min and centrifugation for 10 min at 3200 × g and 4 °C. The GR bound fraction of [1,2,4,6,7–^3^H]-dexamethasone in supernatants was measured by scintillation counting.

#### Experiments in cell culture

Human alveolar carcinoma cell line, A549, the human embryonic kidney cell line, HEK293 and the clonal line of *Spodoptera frugiperda*, SF9 were obtained from the European Collection of Cell cultures (ECACC; distributor Sigma-Aldrich Co.). Cells were grown and maintained in Dulbecco’s modified Eagle’s medium (DMEM, Lonza Group Ltd., Basel, Switzerland) supplemented with glucose (4.5 g/L), heat-inactivated fetal bovine serum (HI-FBS) (10% v/v), penicillin (100 IU/mL), streptomycin (100 µg/mL) and L-glutamine (2 mM). Cells were maintained and grown in a humidified atmosphere (95% air, 5% CO_2_, 37 °C). Unless otherwise stated cells were seeded at 2 × 10^5^ per 35-mm well. Cells were cultured in steroid-free medium for 24 h prior to experimentation. Plasmids were a kind gift from K.E.Chapman, Centre for Cardiovascular Science, University of Edinburgh.

To study GR translocation, HEK293 cells were transfected with GR labelled with Green Fluorescent Protein (GFP-GR). After seeding and overnight incubation in steroid free medium, the medium was replaced with phenol red free Opti-MEM (Lonza Group Ltd., Basel, Switzerland), and cells transfected using Lipofectamine 2000 (Invitrogen, Thermo Fisher Scientific Co., Waltham, MA, USA) with 1 μg of GFP-GR plasmid. Cells were then treated with vehicle, cortisol (1 μM) or 20β-DHF (1 μM) and imaged using fluorescence microscopy (Nikon Eclipse TS100) prior to treatment and at 30, 60, 120 minutes and 4 hours.

GR activation by 20β-DHF was tested in A549 cells transiently transfected with MMTV-luciferase plasmid. Cells were transfected with 1 μg of pMMTV LTR–luciferase^12^ and 1 μg of pKC275 (encoding β-galactosidase as internal control) and treated with vehicle, cortisol (0.3 μM-5 μM) or 20β-DHF (0.3 μM-5 μM) for 4 hours. Cells were lysed and luciferase and β-galactosidase activities measured as described previously^55^. Galactosidase activity was assayed using a Tropix Kit (Applied Biosystems, Foster City, CA, USA). The mean ratio of luciferase/b-galactosidase activities was calculated.

To determine effects of 20β-DHF on endogenous glucocorticoid-induced transcripts, A549 cells were incubated in the presence of increasing concentrations of either cortisol, 20β-DHF (0.15 μM-5 μM) or vehicle (ethanol) for 4 hours. RNA was extracted and RT-qPCR used to quantify *DUSP1* (dual specificity phosphatase 1), *GILZ* (glucocorticoid-induced leucine zipper), *IGFBP1* (insulin-like growth factor binding protein 1) and of *FKBP51* (FK506-binding protein 51) relative to 18 S (see supplementary Table S6 for primer design and supplementary methods). To determine the effects of 20β-DHF on inflammatory transcripts cells were pre-incubated with vehicle or TNFα (10 ng/mL) for 1 hour before a further 4 hours in the presence of vehicle, cortisol or 20β-DHF (0.15 μM–5 µM). RNA was extracted and IL-1β (interleukin 1β) mRNA quantified relative to 18S.

#### MARCoNI analysis of 20β-DHF binding to GR

A microarray Assay for Real-time Co-regulator-Nuclear Receptor Interaction (MARCoNI) was used to compare the quantitative and qualitative co-regulator recruitment induced when 20β-DHF (1 μM) binds with GR with that of recruitment in response to dexamethasone (1 μM) using a previously described method^22, 56^.

### Pharmacological inhibition of CBR1 *in vivo*

For *in vivo* studies in mice, experiments were approved by the University of Edinburgh ethical committee and performed under the Provisions of the Animals Scientific Procedures Act (1986) of the UK Home Office, in accordance with EU Directive 2010/63. Male C57BL/6 J mice aged 8 weeks were purchased from Harlan laboratories and used to conduct a randomized vehicle controlled experiment. Mice were randomly assigned to the vehicle (n = 12) or quercetin-treatment group (n = 12). All the mice were fed ad-lib high-fat diet (D12331, Research Diets inc., New Jersey, USA) for 6 weeks. Quercetin treatment was administered in drinking water (50 μg/mouse/day^57^). Bodyweight, food and water intake were monitored weekly. At week 6 blood collected from tail nick at 0800 h and at 2000h for analysis of plasma basal corticosterone by Enzo Corticosterone EIA Kit (Enzo Life Sciences, Exeter, UK). Mice were fasted for 6 h (0800–1400 h) in clean cages before undergoing a glucoses tolerance test (GTT). Glucose (2 mg/g body weight, 40% w/v in saline) was administered via intraperitoneal injection. Blood was collected from tail nick immediately prior to injection, 15, 30, 60, and 90 minutes after injection. Glucose was measured immediately using a point-of-care glucometer. Plasma insulin was measured using the Ultra-Sensitive Mouse Insulin ELISA kit (Crystal Chem, Inc., IL, USA). Seven days after the GTT animals were culled by decapitation. Plasma was extracted from trunk blood and stored at −20 °C. Tissue was extracted and stored at −80 °C. mRNA and glucocorticoid extraction and quantification are described in supplementary methods (see Supplementary Table S8 for murine primer sequences).

### Statistical analysis

For horse, human and mouse studies data were tested for normality by Kolmogorov-Smirnov test and subsequent comparisons (lean v obese) performed using unpaired Student’s t-tests or Mann-Whitney U test. For cell-based studies with changing steroid concentrations comparisons (20β-DHF v cortisol) were performed using two-way ANOVA with Bonferroni post-hoc tests. Statistical significance was set at P < 0.05.

## Electronic supplementary material

Morgan et al_SupplementaryInfo
