# Supplementary material for: Carbonyl reductase 1 catalyzes 20β-reduction of glucocorticoids, modulating receptor activation and metabolic complications of obesity
Source: Sci Rep. 2017 Sep 6;7:10633. doi: 10.1038/s41598-017-10410-1 (PMC5587574; doi:10.1038/s41598-017-10410-1)
Supplement: Supplementary file 1 — Morgan et al_SupplementaryInfo [file 41598_2017_10410_MOESM1_ESM.pdf]

Carbonyl reductase 1 catalyzes 20β-reduction of glucocorticoids, modulating receptor activation and metabolic complications of obesity

Authors: Ruth A Morgan[1,2], Katharina R Beck[3], Mark Nixon[1], Natalie ZM Homer[4], Andrew A Crawford[1,5], Diana Melchers[6], René Houtman[6], Onno C Meijer[7], Andreas Stomby[8], Anna J Anderson[1], Rita Upreti[1], Roland H Stimson[1], Tommy Olsson[8], Tom Michoel[9], Ariella Cohain[10], Arno Ruusalepp[11,12,13], Eric E. Schadt[10], Johan L. M. Björkegren[10,11,12,13,14], Ruth Andrew[1,4], Christopher J Kenyon[1], Patrick WF Hadoke[1], Alex Odermatt[3], John A Keen[2], and Brian R Walker[1,4,*]

Affiliations:

[1] University/BHF Centre for Cardiovascular Science, The Queen's Medical Research Institute, University of Edinburgh, UK

[2] Royal (Dick) School of Veterinary Studies, University of Edinburgh, UK

[3] Division of Molecular and Systems Toxicology, Department of Pharmaceutical Sciences, University of Basel, Switzerland

[4] Mass Spectrometry Core Laboratory, Wellcome Trust Clinical Research Facility, The Queen's Medical Research Institute, University of Edinburgh, UK

[5] School of Social and Community Medicine, University of Bristol, UK

[6] PamGene International, Den Bosch, The Netherlands

[7] Department of Internal Medicine, Division Endocrinology, Leiden University Medical Center, Leiden, The Netherlands

[8] Department of Public Health and Clinical Medicine, Umeå University 901 87 Umeå, Sweden

[9] The Roslin Institute, University of Edinburgh, Easter Bush Campus, UK

[10]Department of Genetics and Genomic Sciences, Icahn Institute for Genomics and Multiscale Biology, Icahn School of Medicine at Mount Sinai, New York, USA

[11]Department of Physiology, Institute of Biomedicine and Translation Medicine, University of Tartu, Estonia

[12]Clinical Gene Networks AB, Stockholm, Sweden

[13]Department of Cardiac Surgery, Tartu University Hospital, Tartu, Estonia

[14]Integrated Cardio Metabolic Centre, Department of Medicine, Karolinska Institute, Sweden

***Author to whom correspondence should be addressed:**

Professor Brian Walker, Email: b.walker@ed.ac.uk, University/BHF Centre for Cardiovascular Science, The Queen's Medical Research Institute, University of Edinburgh, 47 Little France Crescent, Edinburgh EH16 4TJ

Supplementary Materials:

SM1 Quantification of urinary glucocorticoid metabolites

Glucocorticoids were extracted from equine urine (20mL) by solid phase extraction on Bond Elut Nexus mixed mode Large Reservoir Capacity, 60 mg columns (Agilent Technologies, Santa Clara, CA, USA). Glucocorticoids were extracted from human urine (10mL) by solid phase extraction on Sep-Pak columns (Waters, Milford, MA, USA).

Steroid conjugates were hydrolysed using β-glucuronidase followed by re-extraction. The steroids obtained were derivatized to form methoxime-trimethylsilyl (MO-TMS) derivatives. Steroidal derivatives were separated by gas chromatography using the TRACE GC Ultra Gas Chromatograph (Thermo Fisher Scientific). Analysis was performed on a TSQ Quantum Triple Quadrupole GC-tandem mass spectrometer (Thermo Fisher Scientific) using a 35HT Phenonemex column (30m, 0.25mm, 0.25µm, Agilent Technologies) as previously described (*45, 46*). Epi-cortisol and epi-tetrahydrocortisol were used as internal standards (Steraloids, Newport, RI, USA). The steroids analyzed were cortisol (F), cortisone (E), 5β-tetrahydrocortisol (5β-THF), 5β-tetrahydrocortisone (5β-THE), 5α-tetrahydrocortisol (5α-THF) (*45, 46*) with the inclusion of the following transitions (collision energy) α-cortol (535→355, 20V) and β-cortol (535→455, 10V), α- and β-cortolone (449→269, 10V), 6β-hydroxycortisol (693→513, 10V), 20α- dihydrocortisol (578→488, 10V) and 20β-dihydrocortisol (681→578, 10V).

Steroid quantities in equine urine were expressed as a ratio to creatinine, which was measured using a colorimetric method based on the modified Jaffe's reaction (IL650 analyser, Instrumentation Laboratories, Barcelona, Spain).

SM2 Quantification of glucocorticoids in plasma

Plasma samples (1 mL Equine, 200 µL Human) enriched with internal standard (D4-F, D4-E and D8-B; 250 ng of each) were extracted by liquid-liquid extraction. Chloroform (10 volumes) was added to each sample, mixed and the organic layer was dried under nitrogen (60 °C). The extracts were re-suspended in mobile phase (60 µL, water: methanol 70:30 v/v) for quantification of steroids by LC-MS/MS. The injection volume was 30 µL.

SM3 Quantification of glucocorticoids in adipose tissue

Adipose samples (100mg) were homogenized in ethyl acetate (1 mL) and enriched with internal standard (D4-F, D8-E, D8-B; 250ng of each). The homogenate was slowly dripped onto chilled ethanol: glacial acetic acid: water (95:3:2 v/v, 10 mL) and frozen at -80 °C overnight. Samples were thawed (4 °C) prior to sonication (8 x 15 second bursts) and centrifugation (3000 x g, 30 mins, 4 °C). The supernatant was dried under nitrogen (60 °C), re-suspended in methanol (10 mL) and frozen at -80 °C overnight. Samples were thawed (RT) and hexane (10 mL) added and mixed. The hexane layer was removed, the remaining methanol dried down under nitrogen (60 °C) and re-suspended in water (400 µL) and ethyl acetate (4 mL). The organic layer was removed, dried under nitrogen (60 °C) and re-suspended in 30 % methanol (5 mL). C18 Bond Elut columns (Agilent Technologies, Santa Clara, CA, USA) were conditioned (methanol 5 mL) and equilibrated (water 5 mL), samples were loaded and steroids eluted with methanol (2 mL). Eluates were dried down under nitrogen (60 °C) and re-suspended in 60 µL mobile phase (water: methanol, 70:30 v/v) for quantification of steroids by LC-MS/MS. Injection volume was 30 µL.

SM4 Quantification of mRNA by RT-qPCR

Total RNA was extracted from adipose and liver using the RNAeasy Mini Kit (Qiagen Inc, Valencia, CA, USA). The tissue was mechanically disrupted in either QIAzol (Qiagen) for

adipose tissue or RLT buffer (Qiagen) for liver tissue. Total RNA was extracted from cells in QIAzol lysis reagent using an RNeasy Mini Kit according to the manufacturer's instructions.

Quantitative real-time polymerase chain reaction was performed using a Light-cycler 480 (Roche Applied Science, Indianapolis, IN, USA). Primers were designed using sequences from the National Centre of Biotechnological Information and the Roche Universal Probe Library (see Table S7-9) for details of primers for genes of interest and housekeeping genes). Samples were analysed in triplicate and amplification curves plotted (y axis fluorescence, x axis cycle number). Triplicates were deemed acceptable if the standard deviation of the crossing point was < 0.5 cycles. A standard curve (y axis crossing point, x axis log concentration) for each gene was generated by serial dilution of cDNA pooled from different samples and fitted with a straight line and deemed acceptable if reaction efficiency was between 1.7 and 2.1.

Supplementary Tables and Figures

Table S 1 Characteristics of equine study subjects

	Lean (n=14)	Obese (n=14)
**Age (years)**	15.6 ± 5.6	13.8 ± 7.8
**Sex**	4 Females<br>10 Castrated males	8 Female<br>5 Castrated males
**Breeds**	11 Thoroughbred<br>2 Native pony<br>1 Percheron	2 Thoroughbred<br>3 Cob<br>7 Native ponies<br>1 Arab pony<br>1 Clydesdale
**Body condition score (/5)**	2.3 ± 0.3	3.8 ± 0.7*

Data are expressed as mean ± SEM. Student's t-test or Mann-Whitney U test: *p<0.05

Table S 2 Characteristics of obese human participants with type 2 diabetes providing 24 hour urine samples

**n**	19
**Age (years)**	58.9 ± 1.5
**Body Mass Index (kg/m^2)**	32.60 ± 1.2
**Concurrent medications**	4 no medication<br>15 metformin

All participants were male and diagnosed with diabetes. Participants provided 24 hour urine samples. The additional lean and obese participants providing urine were recruited as part of a different study (Upreti et al 2014). Data are expressed as mean ± SEM.

Table S 3 Characteristics of lean and obese study participants providing plasma samples

	**Lean (n=10)**	**Obese (n=10)**
**Age (years)**	50.5 ± 10.4	50.0 ± 11.8
**Body Mass Index (kg/m^2)**	23.8 ± 1.2	32.9 ± 2.7
**Concurrent mediciations**	• 6 No medication<br>• 1 perindopril, nifedipine,<br>• 1 citalopram, simvastatin, clopidogrel, ranitidine<br>• 1 clomipramine<br>• 1 ranitidine	• 8 No medication<br>• 1 tamsulosin, lansoprazole<br>• 1 pantoprazole

Plasma was collected (between 8 and 9am) from lean and obese but otherwise healthy men. Participants with diabetes or prior corticosteroid treatment were excluded prior to recruitment. Data are expressed as mean ± SEM.

Table S 4 Characteristics of lean and obese men providing adipose biopsy samples during surgery.

	**Lean (n=8)**	**Obese (n=8)**
**Age (years)**	55.7 ± 12.7	52.8 ± 13.7
**Body Mass Index** (kg/m^2)	23.0 ± 1.7	38.8 ± 6.7
**Surgery**	• Cholecystectomy<br>• Laparoscopic cholecystectomy<br>• Removal of gastric band<br>• Hernia repair<br>• Open cholecystectomy<br>• 3 Abdominal hernia repairs	• Gastric bypass<br>• Laparoscopic sleeve gastrectomy<br>• Laparoscopic cholecystectomy<br>• Cholecystectomy<br>• 2 Laparoscopic fundoplications<br>• Laparoscopic cholecystectomy<br>• Laparoscopic removal of gastric band and gastric bypass

All the participants were male. Samples were obtained at the time of surgery. Data are expressed as mean ± SEM.

Table S 5 Estimates of the phenotypic associations with CBR1 expression in the liver based on Mendelian Randomisation

**Outcome**	**Beta**	**Se**	**P value**	**N**	**Sample**
BMI	-0.01	0.01	0.25	339224	(55)
Body Fat	-0.01	0.01	0.06	100716	(56)
2hr glucose adjusted for BMI	<0.01	0.03	0.89	15234	(57)
Fasting glucose	0.01	<0.01	0.02	58074	(58)
Fasting insulin	<0.01	<0.01	0.45	51750	(58)
HOMA-B	<0.01	<0.01	0.59	46186	(59)
HOMA-IR	0.01	0.01	0.27	46186	(59)
HbA1c	0.01	<0.01	0.01	46368	(60)

These analyses used rs1005696 as the instrument for CBR1 expression in liver and associations were analysed using MR-base. See supplementary references for further details of studies used.

Table S 6 Human primer sequences for PCR

**Gene Symbol, full name**	**Forward Primer (3'→ 5')**	**Reverse Primer (5'→ 3')**
RNA18s *(*ribosomal RNA 18s)	CTTCCACAGGAGGCCTACAC	CGCAAAATATGCTGGAACTTT
DUSP1 (dual specificity phosphatase 1)	TTCAAGAGGCCATTGACTT	CCTGGCAGTGGACAAACAC
GILZ (glucocorticoid-induced leucine zipper)	CCGTTAAGCTGGACAACAGTG	ATGGCCTGTTCGATCTTGTT
FKBP51 (FK506-binding protein 51)	GGATATACGCCAACATGTTCAA	CCATTGCTTTATTGGCCTCT
IGFBP1 (insulin-like growth factor binding protein 1)	GCCTTGGCTAAACTCTCTACGA	CCATGTCACCAACATCAAAAA
IL-1β (Interleukin 1β)	TGTAATGAAAGACGGCACACC	TCTTCTTTGGGTATTGCTTGG
CBR1 (Carbonyl Reductase 1)	TCCCTCTAATAAAACCCCAAGG	GGTCTCACTGCGGAACTTCT

Table S 7 Equine primer sequences for PCR

**Gene Symbol, full name**	**Forward Primer (3'→ 5')**	**Reverse Primer (5'→ 3')**
RNA18s *(*ribosomal RNA 18s)	TGACCCAAGGCTAGTAGCTGA	TTCAACACATCACCCACCAT
SDHA (Succinate	CTACGGAGACCTTAAGCATCTGA	GGGTCTCCACCAGGTCAGTA

dehydrogenase complex)		
Equine CBR1 (Carbonyl Reductase 1)	ACCCAGCCATGTCTTACACC	CAGGATAGTGAAGCCGATGC

Table S 8 Murine primer sequences for PCR

**Gene Symbol, full name**	**Forward Primer (3’→ 5’)**	**Reverse Primer (5’→ 3’)**
RNA18s (ribosomal RNA 18s)	CTCAACACGGGAAACCTCAC	CGCTCCACCAACTAAGAACG
Tbp (TATA-binding protein)	GGGAGAATCATGGACCAGAA	GATGGGAATTCCAGGAGTCA
Per1 *(Period 1)*	GCTTCGTGGACTTGACACCT	TGCTTTAGATCGGCAGTGGT
Pepck (phosphoenolpyruvate carboxykinase)	GAGGCACAGGTCCTTTTCAG	GTTCCTGGGCCTTTGTGAC
Adipq *(Adiponectin)*	GGTGAGAAGGGTGAGAAAGGA	TTTCACCGATGTCTCCCTTAG
Lpl *(Lipoprotein lipase)*	CTCGCTCTCAGATGCCCTAC	GGTTGTGTTGCTTGCCATT
Sgk1 *(serum glucocorticoid kinase 1)*	TTTCCAAAGGGGGATGCT	TGTTGGCATGATTACATTGTTCT
ENaC1 *(Epithelial sodium channel 1)*	AGCACAGAGAACACCCCTGT	TGGCTCTTCCTACCCTCTCTC
Cbr1 (Carbonyl Reductase 1)	AGGTGACAATGAAAACGAACTTT	GGACACATTCACCACTCTGC

Figure S 1 The human urinary glucocorticoid metabolite profile in lean and obese individuals

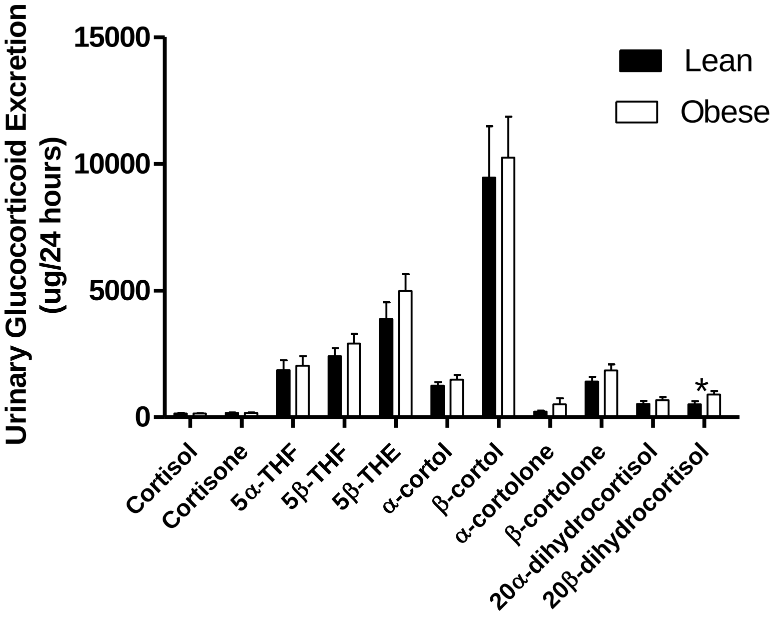

Urinary glucocorticoids of lean and obese participants were extracted, derivitized and quantified by GC-MS/MS. Urinary excretion of 20β-dihydrcortisol was higher in the obese group.

Figure S 2 Equine liver cytosol metabolises cortisol to 20β-dihydrocortisol and this production is inhibited by quercetin

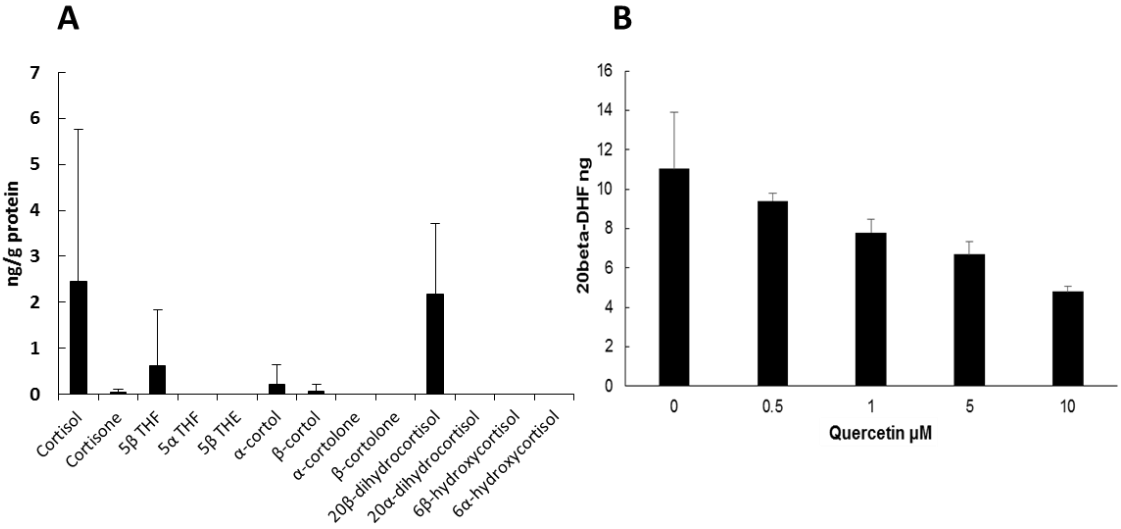

[A] In the presence of NADPH equine liver cytosol produced 20β-DHF as the predominant metabolite. (THF = tetrahydrocortisol, THE = tetrahydrocortisone). [B] CBR1 inhibitor quercetin prevented production of 20β-DHF by equine liver cytosol. Data are mean ± SEM.

Figure S 3 20β-Dihydrocorticosterone is present in murine tissues

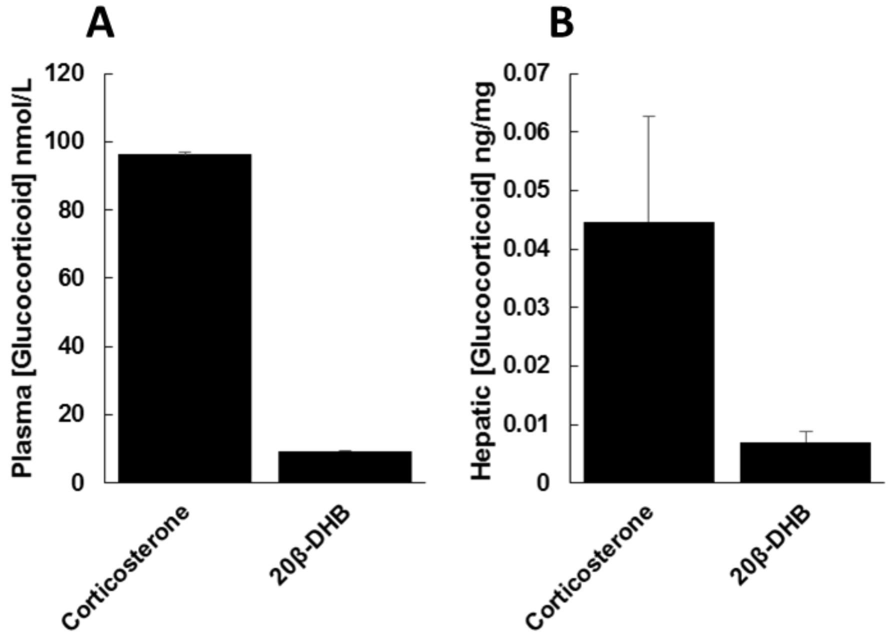

20β-Dihydrocortisone (the murine equivalent of 20β-dihydrocortisol) was measured by LC-MS/MS in murine [A] plasma and [B] liver. Data are mean ± SEM.

Figure S 4 20β-Dihydrocorticosterone induces murine glucocorticoid receptor activation

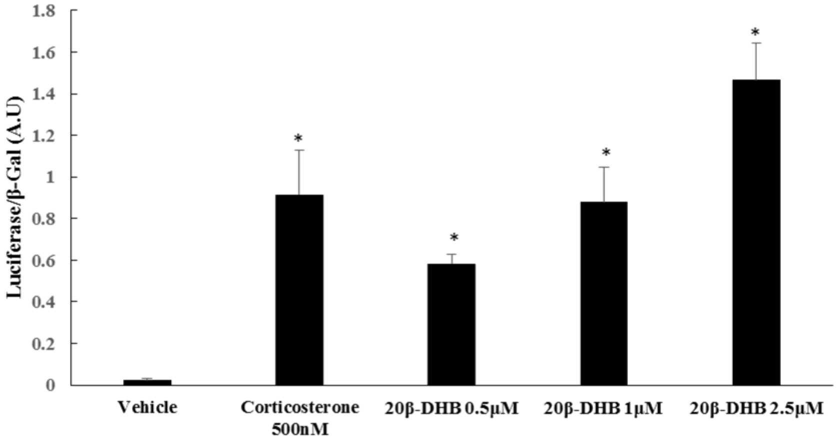

HEK293 cells transfected with murine GR-MMTV-Luc were incubated with increasing concentrations of 20β-dihydrocorticosterone (20β-DHB) the murine equivalent of 20β-dihydrocortisol. Data are mean ± SEM, *P<0.05 compared to vehicle.

Supplementary References

Locke AE, Kahali B, Berndt SI, Justice AE, Pers TH, Day FR, Powell C, Vedantam S, Buchkovich ML, Yang J, et al. Genetic studies of body mass index yield new insights for obesity biology. *Nature.* 2015;518(7538):197-206.

Lu Y, Day FR, Gustafsson S, Buchkovich ML, Na J, Bataille V, Cousminer DL, Dastani Z, Drong AW, Esko T, et al. New loci for body fat percentage reveal link between adiposity and cardiometabolic disease risk. *Nat Commun.* 2016;7(

Saxena R, Hivert MF, Langenberg C, Tanaka T, Pankow JS, Vollenweider P, Lyssenko V, Bouatia-Naji N, Dupuis J, Jackson AU, et al. Genetic variation in GIPR influences the glucose and insulin responses to an oral glucose challenge. *Nat Genet.* 2010;42(2):142-8.

Manning AK, Hivert MF, Scott RA, Grimsby JL, Bouatia-Naji N, Chen H, Rybin D, Liu CT, Bielak LF, Prokopenko I, et al. A genome-wide approach accounting for body mass index identifies genetic variants influencing fasting glycemic traits and insulin resistance. *Nat Genet.* 2012;44(6):659-69.

Dupuis J, Langenberg C, Prokopenko I, Saxena R, Soranzo N, Jackson AU, Wheeler E, Glazer NL, Bouatia-Naji N, Gloyn AL, et al. New genetic loci implicated in fasting glucose homeostasis and their impact on type 2 diabetes risk. *Nat Genet.* 2010;42(2):105-16.

Soranzo N, Sanna S, Wheeler E, Gieger C, Radke D, Dupuis J, Bouatia-Naji N, Langenberg C, Prokopenko I, Stolerman E, et al. Common variants at 10 genomic loci influence hemoglobin A1C levels via glycemic and nonglycemic pathways. *Diabetes.* 2010;59(12):3229-39.
